# The task dependent differences in electromyography activity of hamstring muscles during leg curls and hip extensions

**DOI:** 10.1371/journal.pone.0245838

**Published:** 2021-02-09

**Authors:** Norikazu Hirose, Yoshinori Kagaya, Masaaki Tsuruike

**Affiliations:** 1 Faculty of Sport Sciences, Waseda University, Nishitokyo, Tokyo, Japan; 2 Department of Physical Therapy, Showa University, Yokohama, Kanagawa, Japan; 3 Department of Kinesiology, San Jose State University, San Jose, California, United States of America; University of Pittsburgh, UNITED STATES

## Abstract

This study aimed to investigate the influence of the task type on the relative electromyography (EMG) activity of biceps femoris long head (BFlh) to semitendinosus (ST) muscles, and of proximal to distal regions during isometric leg-curl (LC) and hip-extension (HE). Twenty male volunteers performed isometric LC with the knee flexed to 30° (LC30) and 90° (LC90), as well as isometric HE with the knee extended (HE0) and flexed to 90° (HE90), at 40% and 100% maximal voluntary contraction (MVIC). Hip position was neutral in all conditions. EMG activity was recorded from the proximal and distal region of the BFlh and ST muscles. BFlh/ST was calculated from the raw root-mean-square (RMS) amplitudes. The RMS of 40% MVIC was normalized using MVIC data and the proximal/distal (P/D) ratio of normalized EMG (NEMG) was calculated. The BFlh/ST ratio was higher in HE0 than in LC90 during MVIC and 40% MVIC (p<0.05), and was higher in HE90 than in LC90 (p<0.05) during 40% MVIC at the proximal region, whereas no difference was observed between HE0 and LC30. There was no inter-task difference in BFlh/ST ratio in the distal region. Furthermore, the P/D ratio was higher in LC90 than in LC30 and HE0 (p<0.05) in BFlh and ST muscles, and was higher in HE90 than in LC30 and HE0 (p<0.05) in BFlh during 40% MVIC. However, there was no difference in P/D ratio between LC30 and LC90, and HE0 and HE90. This showed that there was no task-dependent difference in the EMG activity of the BFlh muscle relative to the ST muscle between prone hip extension and prone knee flexion when the knee joint was set at an equivalent angle. Similarly, there was no task-dependent difference in the NEMG of the proximal region relative to the distal region in BFlh and ST muscles during 40% MVIC.

## Introduction

The hamstrings, which are located in the posterior compartment of the thigh, are composed of four muscles: the biceps femoris long head (BFlh), semimembranosus (SM), semitendinosus (ST), and biceps femoris short head muscles [1]. Of these muscles, the BFlh muscle is known to be most susceptible to hamstring strain injury during sports activities [2–4]. Moreover, hamstring strain injury has a high recurrence rate [5,6]. In order to develop proper exercise protocols to reduce hamstring strain injury, a detailed understanding of the activity of each of the hamstring muscles during any type of movement is warranted. Therefore, a number of studies have investigated the activation of the hamstring muscles, especially the BFlh and ST muscles, using surface electromyography (EMG) [7–15] or magnetic resonance imaging (MRI) [14–19].

The BFlh and ST muscles, as a bi-articular muscle, are responsible for both knee flexion and hip extension (HE) [1]. However, the EMG activity of the biceps femoris muscle relative to the medial hamstring (MH) muscle, which consists of the ST and SM muscles, was lower than that obtained during 45° HE and lunge exercises [8]. This result indicates that the MH muscle works more during knee-dominant movement. This seems to be aligned with muscle functional MRI results as summarized by Bourne et al. [7] Similarly, a study of high-density EMG, taking regional differences into account, also showed higher ST-to-BFlh activity ratio in the knee-dominant Nordic hamstring exercise than in the hip-dominant stiff-leg deadlift (SDL) [13].

The consensus has not been reached whether the EMG activity of the BFlh is higher than the ST muscle during hip-dominant movement. A few studies have reported that the EMG activity of the biceps femoris muscle is higher than that of the ST muscle during SDL [15], and that of the MH muscle during HE [8]. This means that the EMG activity of the biceps femoris muscle is higher during hip-dominant movement than during knee-dominant movement compared to the ST muscle. Correspondingly, the activation ratio of the biceps femoris muscle relative to the MH muscle was higher during HE than during leg curl (LC) [8]. However, Watanabe et al. reported that there was no difference in the EMG activity of the BFlh muscle between prone HE and prone knee flexion [9]. One possible mechanism for this discrepancy might be the difference in the joint angle which influences the anatomical muscular length. Watanabe et al. adopted knee flexion and HE at a knee flexion angle of 0°, which meant that the biarticular hamstring muscles were extended. On the other hand, the hamstring muscles shorten during LC, which was adopted as the knee-dominant movement, whereas hamstrings are set at the stretched position during SDL and HE [8,14,15]. The difference in the knee joint angle, which alters the muscular length, may modulate the EMG activity of the hamstring muscles. For example, some studies suggest that the EMG activity of the BFlh is the highest at a relatively more extended knee position (0-60°) as compared to the ST (60-120°) [10,11].

Recent studies have focused on the regional difference in the EMG activity of these muscles. The ST muscle is divided into proximal- and distal portions with respect to the fibrous septum, and the fascicular length is almost the same between the regions [1,20]. Moreover, most of the ST muscle is innervated by two motor branches from the sciatic nerve, with each branch supplying the proximal and distal regions of the muscle belly [1]. Contrarily, the branch of the motor nerve of the BFlh muscle terminates proximal to the middle region of the muscle belly [1,20–22]. Based on this anatomical difference between the BFlh and ST muscles, several studies have investigated the regional differences in muscular activity of these muscles using MRI and EMG. However, this phenomenon has not been fully understood. For instance, Kubota et al. reported that the ST muscle works more at the middle to the proximal region during LC. In contrast, no difference was reported in the BFlh muscle between pre and post exercise using the transverse relaxation time (T2) [17]. On the other hand, Mendeguchia demonstrated that a significant difference was observed in the proximal region of the BFlh muscle after performing lunge exercise, whereas no regional difference was observed in the short tau inversion recovery value of the ST muscle after performing LC [18]. Moreover, EMG activity at the distal region of the biceps femoris and ST muscles was higher during LC than during lunge exercise, whereas there was no difference in EMG activity at the proximal portion of these muscles during LC and SDL [19]. However, Hegyi et al. reported that EMG activity at the middle portion of the ST muscle was higher than that at its proximal and distal portions during Nordic Hamstring exersise and SDL. On the other hand, EMG activity at the distal portion of the BFlh muscle was higher than that at its proximal portion regardless of the movement [13]. These results imply that task-dependent characteristics must be considered for the relative activity of the BFlh to the ST muscles.

Based on previous findings, this study aimed to investigate the effect of task type on the relative electromyography (EMG) activity of the biceps femoris long head (BFlh) to the semitendinosus (ST) muscle at the proximal and distal region during isometric leg-curl (LC) and hip-extension (HE). In addition to the BFlh and ST muscles, the EMG activity of the gluteus maximus was measured to confirm the compensation of the gluteus maximus during exercise. Furthermore, this study investigated the EMG activity of proximal regions relative to distal regions in BFlh and ST muscles across each of the movements. This study hypothesized that during open kinetic isometric movements, the EMG activity of the BFlh relative to the ST muscles would differ between leg-curls and hip-extensions. We further hypothesized that either the ST or BFlh muscles would demonstrate proximal/distal (P/D) region ratio differences in EMG activity across various movements.

## Methods

### Study design

**Fig 1:**
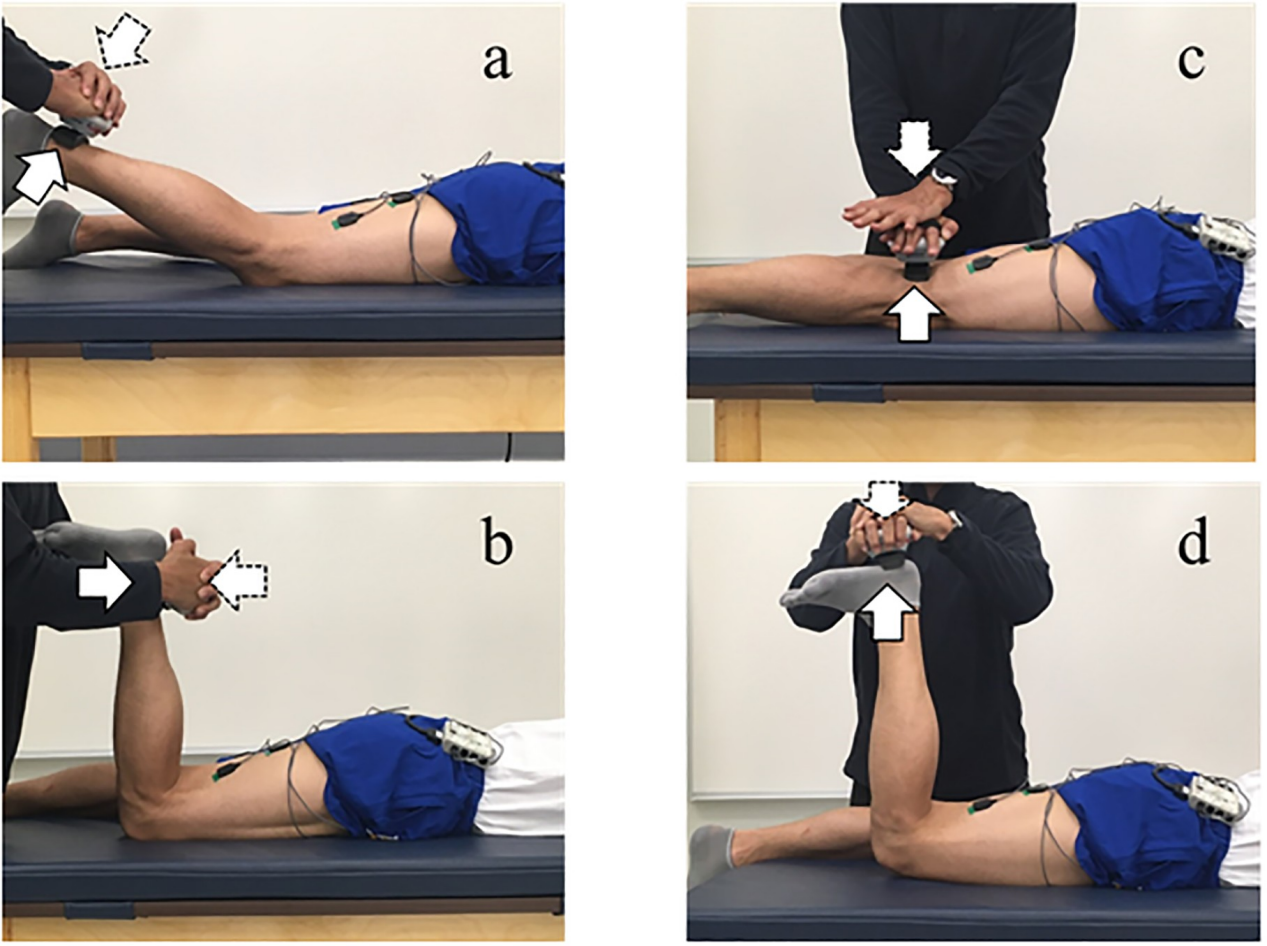
Leg curl at 30° knee flexion (a), leg curl at 90° knee flexion (b), hip extension with extended knee angle (c), and hip extension with flexed knee angle (d). All movements were performed at MVIC and at 40% of MVIC. Note: The solid arrow indicates the direction of force produced by the subject. The broken arrow indicates the direction of force applied by the examiner. The hip joint was set at the neutral position in the frontal and sagittal planes during all trials. The examiner asked subjects to maintain their ankle in neutral (90°) position.

This research was designed as a case-control study. Participants performed isometric leg curl at 30° and 90° knee flexion angles (LC30, LC90), and hip extensions with the knee extended (HE0) and flexed to 90° (HE90) in the prone position with maximum voluntary isometric contraction (MVIC) and 40% of MVIC (40% MVIC). During LC, the knee flexion angle was randomly set at 30° and 90° with the neutral hip position. During HE0 and HE90, the hip flexion angle was set at the neutral position, and the knee flexion angle was set at 0° and 90°, respectively (Fig 1). EMG data was recorded for the gluteus maximus muscle in addition to the BFlh and ST muscles during each movement. EMG data was collected and analyzed from both the distal and proximal portions of the ST and BFlh muscles.

### Participants

At first, we recruited 21 male volunteers from college sports club teams. The potential participants were excluded if they had a history of hamstring strain injury and anterior cruciate ligament injury at the measured limb or a recent history of lower limb injury within 6 months prior to the experiment. After applying these exclusion criteria, 20 male collegiate volunteers (mean age: 20.4 ± 1.0 years; height: 1.76 ± 0.05 m, body mass: 73.0 ± 6.8 kg) participated in this study. The participants were engaging sporting activity (10 judo, 6 weight training, 4 track and field) at least 3 times per week. All participants had experienced hamstring training, such as leg curl, hip bridge, and Nordic Hamstring exercise. All study protocols were approved by the institutional review board of San Jose State University (F16079). This study conforms to the Declaration of Helsinki. All subjects were fully informed of the procedures and the purpose of this study and provided written informed consent. The participants were free to withdraw from participation at any time without fear of consequences.

### Procedure

Before testing, all participants performed 5 minutes of warm-up exercises, such as static stretching of hamstring muscles for 1 minute, mobilizing measured muscles with a foam rolling for 1 minute, dynamic stretching for 2 minutes, and 5 repetitions of LC and 5 repetitions of HE with knee angle set at 0° and 90° to familiarize themselves with the subsequent tests. After warm-up, subjects performed two bouts of 5-second MVIC in four different positions: LC30, LC90, HE0 and HE90. To minimize the sudden increment of force production, which may lead to the unreliability of obtained data by using the manual measurement of maximum force, the examiner instructed participants to increase force production from sub-maximal effort and reach maximum within 3 seconds.

The peak force was measured using a digital handheld dynamometer (MicroFET2; Hoggan, UT, USA). The peak force for each exercise was used to normalize each of the muscle activities in subsequent tests. The digital handheld dynamometer was set at approximately 5 cm proximal from the heel (LC30 and LC90), above of the popliteal fossa (HE0), and the planter area of the heel was in line with the tibia (HE90). The primary examiner instructed participants not to produce knee flexion force during HE0 and HE90. During HE90, the examiner confirmed participants produce only HE0 while vertically applying the amount of force to the subject with the hand-held dynamometer against gravity.

After two bouts of MVIC measurements, the participants performed two bouts of 5-second isometric LC30, LC90, HE0, and HE90, with the manual resistance given at 40% MVIC in a random order in a single day. All participants had at least 1 minute of rest between sets, and 2 minutes of rest between trials. Each exercise was performed using the dominant leg, which was defined as the side with which the participant would kick. Force was monitored by the examiner using a handheld dynamometer, and the participants were asked to match the amount of force given by the examiner.

One of the three examiners confirmed the knee joint angle was set at targeted angle by using a goniometer. On the other hand, the amount of hip joint angles during HE and knee flexion was monitored visually; and the hip joint was set at a neutral position in the frontal and sagittal planes by another examiner. If the joint angle was not set at the appropriate angle during exercise, it was remeasured. Additionally, we asked subjects to maintain their ankle set at a neutral position.

**Fig 2:**
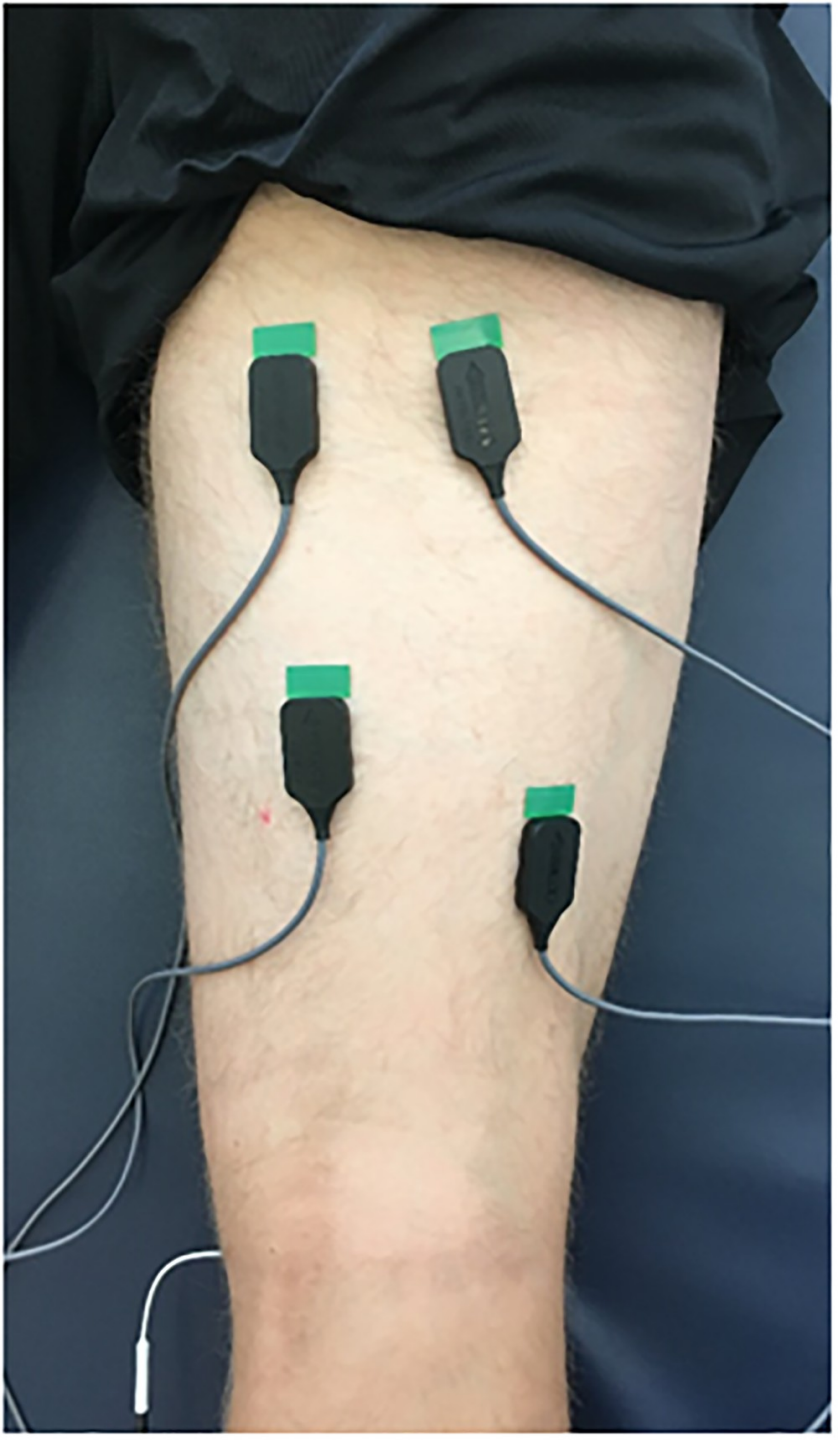
Location of electrodes. The muscle length of BFlh and ST was determined as the length from the ischial tuberosity to the prominence of the lateral- and medial-epicondyle, respectively. The proximal portion of the BFlh and ST were set at 30% of each muscle length distally from the IT. The distal portion of the BFlh and ST were set at 30% and 50% of each muscle length proximally from the lateral- and medial-epicondyle. The examiner palpated the muscle belly of each muscle during the leg curl at 30° and 90°, then confirmed that each of electrode was located on each muscle accurately.

The BFlh, ST, and gluteus maximus muscle EMG activities were measured using bipolar surface electromyogram silver electrodes (Bagnoli-8; Delsys Inc, Natick, MA, USA). The EMG electrodes were pre-amplified (10x) and routed through the EMG mainframe, which further amplified (100x) a total gain of 1000x and band-pass filtered (20–450 Hz) the signals. This study adapted an active EMG electrode with an inter-electrode spacing of 10 mm, length of 10 mm, and diameter of 1 mm. Skin impedance was reduced by shaving the hair at the electrode site and wiping the skin with rubbing alcohol before applying the electrodes. To begin, we measured the BFlh and ST muscle lengths, which were defined as the length from the ischial tuberosity to the prominence of the lateral and medial epicondyle, respectively. As previously reported, the EMG activity of the BFlh muscle is recordable at 25–75% of the longitudinal line of the muscle [9]. Thus, the proximal portion of the BFlh muscle was set at 30% away from the IT, and the distal portion was set at 30% proximally from the lateral epicondyle. On the other hand, previous research reported that the average length of the distal pure tendon of the ST muscle is about 18 cm, equivalent to approximately 40% of the muscle-tendon length, and the proximal muscle-tendon junction is approximately 25% of the muscle-tendon length [23,24]. Thus, the proximal portion of the ST muscle was set at 30% of the ST muscle length distal to the IT, and the distal portion was set at 50% of the ST muscle length proximal to the lateral and medial epicondyle (Fig 2). The electrode was placed at the midpoint between the sacral vertebrae and the greater trochanter for the gluteus maximus muscle. Accurate placement of the electrodes was validated by three skilled athletic trainers as palpation of the muscle bellies. Additionally, the primary examiner confirmed the EMG activity was recorded clearly. This study reduced the root mean square (RMS) from all raw EMG data during the middle 2 seconds of each 5-second exercise for further analysis. Then we calculated the ratio of BFlh to ST activity (BFlh/ST ratio) by dividing the raw EMG activity of the BFlh muscle by the ST muscle value either in the MVIC or 40% MVIC trials. Additionally, we normalized the calculated RMS data during 40% of MVIC using the RMS data of MVIC (normalized EMG [NEMG]) for analyzing the differences of EMG activity between the proximal and distal regions of corresponding muscles. In addition, the EMG of the gluteus maximus during LC30, LC90, and HE was normalized by the values at HE90 (NEMG).

### Statistical analyses

The EMG data obtained during greater force production within two bouts of MVIC trials were analyzed as MVIC condition. In case of the 40% MVIC condition, the EMG data of two bouts of trial were averaged for further analysis. To analyze the reliability of test-retest data, the intraclass correlation coefficients (ICCs) of the force during MVIC and EMG data during 40% MVIC were analyzed for each muscle and each region in each trial. The average values and standard deviation for each condition were calculated. A two-way analysis of variance (ANOVA) design within subjects crossed with loads, and movements was used to identify differences in each mean ratio of BFlh to ST muscle activity at proximal and distal regions. In addition, we analyzed the differences in NEMG during the 40% of MVIC condition between muscles and tasks using two-way ANOVA. Furthermore, the differences between the type of movement and load in terms of the NEMG of the gluteus maximus were analyzed using two-way ANOVA. Where appropriate, the simple main effect and Tukey’s post hoc test were used to measure any differences. Additionally, partial η2 were also analyzed, and the mean difference and 95% confidence intervals (CIs) were reported. Statistical significance was set at p<0.05.

## Results

### ICC

The ICC(1.1) ranged from 0.79 to 0.96 in force during MVIC. Additionally, the ICC(1.1) ranged from 0.86 to 0.95 and 0.82 to 0.94 in the BFlh and ST muscles, respectively, for both the proximal and distal regions. Furthermore, the ICC ranged from 0.19 to 0.98 in the gluteus maximus (Table 1).

**Table 1:** The ICC(1.1) between two bouts of MVIC force and EMG data during 40% MVIC trial.

		LC30	LC90	HE0	HE90
Force during MVIC		0.90	0.83	0.96	0.79
EMG (40% of MVIC)					
BFlh	Proximal	0.91	0.87	0.89	0.91
	Distal	0.86	0.94	0.95	0.92
ST	Proximal	0.83	0.90	0.91	0.94
	Distal	0.82	0.93	0.87	0.84
GM		0.19	0.13	0.98	0.94

BFlh: biceps femoris long head; ST: semitendinosus; GM: gluteus maximus; EMG: electromyography; LC: leg curl; ICC: intraclass correlation coefficient; MVIC: maximum voluntary isometric contraction; HE0: hip extension; HE90: hip extension with knee flexion.

### Force during MVIC

The average force during MVIC was lower (F(3,76) = 10.5, p<0.001) in LC90 (170.3±25.2 N, 95% CI: 158.5–182.1 N) than that of LC30 (218.1±52.5 N, 95% CI: 193.6–242.7 N, p = 0.004), HE0 (228.3±52.3 N, 95% CI: 203.9–252.9 N, p<0.001), and HE90 (240.7±32.9 N, 95% CI: 225.3–256.1 N, p<0.001), whereas there was no difference among other movements.

### BFlh/ST ratio

There was no two-way interaction at the proximal (F(3,114) = 1.5, p = 0.244, partial η2 = 0.090) and distal regions (F(3, 114) = 0.1, p = 0.955, partial η2 = 0.01). However, a significant main effect was observed among different tasks at the proximal (F(3,114) = 8.8, p<0.001, partial η2 = 0.276) and distal (F(3,114) = 2.0, p = 0.015, partial η2 = 0.168) regions. The BFlh/ST ratio of HE0 at the proximal region was higher than that of LC90 in either MVIC (p<0.001) or 40% MVIC (p = 0.011). In addition, the ratio of HE90 at the proximal region was higher than that of LC90 (p = 0.005). In contrast, there was no significant post-hoc difference among tasks at distal region. Furthermore, there was no main effect between loads in either at the proximal (F(1,19) = 0.1, p = 0.875, partial η2 = 0.001) or distal regions (F(1,19) = 0.3, p = 0.615, partial η2 = 0.014) (Table 2).

**Table 2:** The BFlh/ST ratio in proximal and distal regions among movement types at different loads.

BFlh/ST ratio		LC30	LC90	HE0	HE90
Proximal	MVIC	0.80±0.24 (0.68–0.91)	0.63±0.14 (0.56–0.69) HE0	0.93±0.30 (0.79–1.07) LC90	0.74±0.27 (0.61–0.86)
	40% MVIC	0.73±0.19 (0.64–0.81)	0.60±0.21 (0.51–0.70) HE0, HE90	0.87±0.31 (0.72–1.01) LC90	0.87±0.32 (0.72–1.02) LC90
Distal	MVIC	1.23±0.42 (1.04–1.43)	1.00±0.35 (0.83–1.16)	1.34±0.46 (1.13–1.55)	1.23±0.72 (0.89–1.57)
	40% MVIC	1.21±0.40 (1.03–1.40)	0.99±0.39 (0.81–1.17)	1.24±0.53 (0.99–1.48)	1.20±0.53 (0.95–1.45)

LC30, LC90, HE0, and HE90, presented after () indicate a significant difference (p<0.05) between each corresponding movement.

BFlh: biceps femoris long head; ST: semitendinosus; GM: gluteus maximus; EMG: electromyography; LC: leg curl; MVIC: maximum voluntary isometric contraction; HE0: hip extension; HE90: hip extension with knee flexion.

### P/D ratio of NEMG in 40% of MVIC condition

There was no significant two-way interaction (F(3,114) = 1.28, p = 0.284, partial η2 = 0.010). However, a significant main effect was observed among tasks (F(1,114) = 17.9, p<0.001, partial η2 = 0.440), and the P/D ratio of the BFlh muscle during LC90 and HE90 was higher than that during LC30 (p = 0.005, p = 0.006, respectively) and HE0 (p = 0.003 for both). Furthermore, the P/D ratio of the ST muscle was higher in LC90 than in LC30 (p = 0.004) and HE0 (p<0.001). There was no main effect between muscles (F(1,19) = 1.3, p = 0.267, partial η2 = 0.060) (Table 3).

**Table 3:** The P/D ratio of NEMG in BFlh and ST muscles among movement types.

LC30		LC90	HE0	HE90
BFlh	0.86±0.17 (0.78–0.94) LC90, HE90	1.29±0.50 (1.06–1.53) HE0	0.83±0.22 (0.73–0.93) LC90, HE90	1.29±0.57 (1.02–1.56) HE0
ST	0.89±0.22 (0.79–0.99) LC90	1.27±0.44 (1.01–1.48) LC30, HE0	0.76±0.28 (0.63–0.89) LC90	1.04±0.38 (0.86–1.22)

LC30, LC90, HE0, and HE90 presented after () indicate a significant difference (p<0.05) between each corresponding task.

P/D: proximal/distal; NEMG: normalized electromyography; BFlh: biceps femoris long head; ST: semitendinosus; LC: leg curl; HE0: hip extension; HE90: hip extension with knee flexion.

### Gluteus maximus

There was no interaction between load and type of movement (F(2,114) = 1.2, p = 0.315, partial η2 = 0.020). However, a main effect was observed in load (F(1,114) = 5.8, p = 0.018, partial η2 = 0.048), and the NEMG of LC30 in 40% MVIC was higher than that in MVIC (p = 0.040), whereas there was no intra-load difference in other movements. Furthermore, a main effect was observed in type of movement (F(2,114) = 76.5, p<0.001, partial η2 = 0.573), and the NEMG of HE0 was higher than that of LC30 and LC90 in both MVIC and 40% MVIC (all p<0.001) (Table 4).

**Table 4:** The NEMG of GM of LC30, LC90, and HE0 compared to the value of HE90.

	LC30 (%)	LC90 (%)	HE0 (%)
MVIC	20.6±14.2 (14.0–27.3)#,HE0	20.4±17.7 (12.1–28.6)HE0	76.2±20.0 (66.8–85.5)LC30,LC90
40% MVIC	34.2±20.6 (24.5–43.8)#,HE0	33.1±21.2 (23.2–43.0)HE0	77.1±27.9 (64.1–90.2)LC30,LC90

^#^ p<0.05 between MVIC and 40% MVIC. LC30, LC90, and HE0 presented after () indicate a significant difference (p<0.05) between each corresponding movement.

NEMG: normalized electromyography; GM: gluteus maximus; LC: leg curl; HE0: hip extension; HE90: hip extension with knee flexion; MVIC: maximum voluntary isometric contraction.

## Discussion

This study investigated the difference in the BFlh/ST EMG activity ratio at the proximal and distal regions of corresponding muscles between hip extension and knee flexion at different knee flexion angles and loads during isometric contraction. Additionally, we investigated the P/D ratio of NEMG in the BFlh and ST muscles between different tasks at sub-maximum load. In partial support of our hypothesis, the BFlh/ST ratio differed between LC90 and HE0 at the proximal region; however the difference was not observed when the knee joint was set at equivalent angle (i.e., LC30 vs. HE0). Moreover, there was no task-dependent difference observed at the distal region. Additionally, the P/D ratio in LC90 was higher than that in LC30 and HE0 in both BFlh and ST muscles, and the ratio in HE90 was higher than that in LC30 and HE0 in the BFlh during 40% MVIC. However, a P/D ratio difference was observed between LC and HE when the knee joint was set at an equivalent angle. Moreover, there was no inter-muscular difference in the P/D ratio. This result may imply that the P/D ratio does not differ between muscles and tasks when the knee joint is set at an equivalent angle, while the ratio will change across knee joint angles during a task.

The EMG activity of the gluteus maximus during LC30 and LC90 was approximately 20% to 30% compared to that during HE90, and no difference was observed between LC30 and LC90, which indicated that the subject did not compensate for LC movement by using gluteus maximus activity. These data indicate the valid data were obtained in this study.

The BFlh/ST ratio was higher at the proximal region during HE0 than during LC90 in MVIC and 40% MVIC. This result implies that the relative activity of the BFlh to ST muscles at the proximal region will be higher according to the knee flexion movement to hip extension movement, and this result seems to correspond with previous studies [15,16]. However, when the knee joint was set at an equivalent angle and comparing isometric LC30 with HE0, and LC90 and HE90, there was no inter-movement difference in the BFlh/ST activity ratio. This result was inconsistent with previous studies which have suggested that the BFlh muscle is relatively more worked during hip-dominant movement while the ST muscle works more during knee-dominant movement. [7,8,13,15] Possible reasons for this discrepancy include differences in joint angles during tasks. Several studies have reported the inverse relationship of EMG activity between the BFlh and ST muscles across different knee flexion angles during prone LC [10,11] and prone table HE0 [25]. A previous study, which used fine wire electrodes, reported that the NEMG activity of the ST muscle increased, whereas that of the BFlh muscle decreased as the knee flexion angle increased from 0° to 105° [10]. A similar trend regarding the relative EMG activity of the BFlh muscle compared to the ST muscle has been reported, and the activity of the BFlh muscle was higher than the ST muscle at 30° of knee flexion, while the activity of the ST muscle was higher than the BFlh muscle at 90° of knee flexion during isometric prone LC [11]. Furthermore, this inverse relationship in EMG activity between the BFlh and ST muscles was also reported during prone table HE0, in which the NEMG activity of the BFlh muscle relative to the ST muscle was higher (69.37% vs. 36.80%) when the knee angle was set at 0°, whereas the relative EMG activity of the BFlh muscle was comparable to the ST muscle (25.22% vs. 27.60%) [25]. This previous study could not clarify the underlying mechanism of this result. However, the difference has been suggested in morphological features (i.e., shorter fiber length with or without pennation angle) [23,24] and moment arm (i.e., the ST muscle moment arm is maximized with increasing knee flexion angle, whereas it decreases in the BFlh muscle) [15] between the BFlh and ST muscles may be responsible for the influence of joint angle on the variations in the BFlh and ST muscle activities. In contrast, the inverse relationship in EMG activity between the BFlh and ST muscles, which was shown during prone LC and prone HE0, must be altered by kinetic change. For instance, the relative EMG activity of the BFlh muscle to the ST muscle was not changed by altering the knee flexion angle during either bilateral or unilateral hip bridge exercise, which is known as closed kinetic chain movement [11]. Although we need to take the influence of the cross-talk of adjacent muscles into consideration. In the case of the SM muscle, the knee joint angle relationship of the EMG activity during prone LC is inconsistence among previous studies. Different previous studies report that the EMG activity increased [10] or decreased [11] with knee flexion angle increase. Thus, clarifying the characteristics of the activity of the SM muscle across various knee joint angles might help improve our understanding of the task-dependent relationship among the BFlh, ST, and SM muscles. On the other hand, the BFlh/ST ratio was higher in HE90 than that in LC90 at proximal region during 40% MVIC. This result implies that the BFlh/ST ratio may change between tasks even if the knee joint is set at an equivalent angle. Further investigation regarding the mechanism behind this finding is required from applied loads and/or regional characteristics of the muscles.

In support of our hypothesis, there were task-dependent differences in the P/D ratio of the BFlh and ST muscles. However, there was no difference in the P/D ratio between muscles. Moreover, there was no difference in the P/D ratio between tasks when the knee joint was set at an equivalent angle during the 40% MVIC condition. The rationale of our hypothesis was that the ST muscle is partitioned by the fibrous septum into the proximal and distal portions [1,20], and that these regions are innervated by two different primary nerve insertions [1]. We further hypothesized that these morphological characteristics of the ST muscle would contribute to the response of a task-dependent difference in the EMG ratio between the proximal and distal regions. If these morphological characteristics of the ST muscle were responsible for the previous result, the P/D ratio difference should also have been shown in LC30 and HE0 and/or LC90 and HE90. Thus, the rationale of our study could not be responsible for the result. Similarly, we did not observe a task-dependent difference in the P/D ratio in the BFlh muscle when the knee angle was set at an equivalent angle, while a previous study suggested that there is a task-dependent difference (lunge vs. leg curl) in the EMG activity of the BFlh between proximal and distal regions [19]. On the other hand, the P/D ratio changed through alteration to the knee joint angle, and the ratio in LC90 was higher than that in LC30. A similar trend was observed between HE90 and HE0 in the BFlh. This study could not provide an exact reason for the result. One possible mechanism for this result could be the occurrence of muscle shift under the skin during knee flexion, which is often argued as a limitation of surface electrodes. In this study, the distal ST electrode was placed at 50% of the ST muscle length (approximately 22 cm) proximal from the prominence of the medial tibial condyle. A distal ST tendon inserts at the upper part of the medial surface of the tibia, 4 cm distal from the tibial tuberosity [24]; thus, theoretically, the distal ST surface electrode recorded activity 26 cm away from the ST insertion. Because the mean length of the ST pure tendon ranges from 11.1 cm to 17.9 cm [1,26], a distal electrode can record the distal ST EMG activity even though the ST muscle is shortened during muscular contraction by 8 cm. However, Kumazaki et al. reported that averaged total ST muscular length decreased by approximately 7 cm as knee flexion angle increased from 0° (381.5 mm) to 90° (314.5 mm) [27]. Therefore, the distal electrode in this study may have recorded the activity at the muscular tendon junction region during LC90 and HE90. As previously reported, the EMG amplitude decreases at the tendon and innervation zone [28,29], thus, the P/D ratio was higher during LC90 and HE90 compared to that during LC30 and HE0. In addition, the results of our study are limited to only 40% MVIC; therefore, further investigation of other loads is required.

A possible limitation of this study was that we did not identify the proximal and distal portion of the BFlh or ST muscles by ultrasound. During EMG, the location where the electrodes are positioned is critically important [28,29]. Thus, further research confirming the location of the electrode using ultrasound is warranted. Further, comprehensive investigations regarding the difference in EMG activity of the BFlh and ST muscles are required. For instance, possible EMG activity influencers, such as horizontal and frontal joint angle of hip rotation and abduction or adduction [12] and the joint angle of the ankle, still exist. Additionally, the joint angle monitoring procedure had some limitations. We monitored each of the joint angles, during exercises, using a manual goniometer. In the previous study that used EMG, the change of joint angle was not negligible [29]. Thus, further research using advanced methods to monitor joint angles is recommended. In addition, we need further studies including female subjects to generalize the finding of this study. Finally, previous studies divide the MVIC procedure, such as resisting a load, which is eccentric in nature, and contracting against an immovable object, which is concentric in nature, and this study adapted the former procedure [30,31]. However, because the data of both conditions are under the sub-maximum effort [30], we need to keep in mind that our data reflects either isometric or eccentric contraction of corresponding muscles although we describe it as “isometric contraction” through the paper.

The findings of this study may be applicable to sport clinical settings. The fact that the activity of the BFlh muscle relative to the ST muscle did not differ between LC and HE when the knee joint was set at an equivalent angle indicates that performing knee-dominant movements such as prone LC and Nordic Hamstring exercises, will activate the BFlh muscle as a hip-dominant movement such as hip-extension, at least under maximum load. Indeed, a recent study reported that the EMG activity of the BFlh muscle is higher than that of the ST muscle even during Nordic Hamstring exercise at shallow knee flexion angles [32], which is known as the knee-dominant exercise [7]. In addition, previous research suggests that hamstring injuries occur predominantly in the BFlh muscle [33,34], which is most susceptible to injury during the terminal leg swing phase of sprinting [35,36]. Thus, developing BFlh dominant exercises using knee dominant movement at shallow knee flexion angles may be a reasonable way to reduce the risk of initial and/or recurrent hamstring injury.

## Conclusion

There was no task-dependent difference in EMG activity of the BFlh muscle relative to the ST muscle between LC and HE when the knee joint was set at equivalent angle at proximal and distal region, except for at proximal region during 40% MVIC. In addition, there was no task-dependent difference in EMG activity of the proximal relative to distal region in BFlh and ST muscles when the knee joint was set at equivalent angle. In addition, altering knee joint angle may influence on the P/D ratio during leg-curl in BFlh and ST muscles, and during HE in BFlh muscles at 40% MVIC condition.

## Supporting information

S1 File (PDF)

S2 File (PDF)
