# Supplementary material for: The task dependent differences in electromyography activity of hamstring muscles during leg curls and hip extensions
Source: PLoS One. 2021 Feb 9;16(2):e0245838. doi: 10.1371/journal.pone.0245838 (PMC7872291; doi:10.1371/journal.pone.0245838)
Supplement: S2 File — (PDF) [file pone.0245838.s002.pdf]

SJSU SAN JOSÉ STATE UNIVERSITY

Office of Research
Division of
Academic Affairs

San José State University
One Washington Square
San José, CA 95192-0025

TEL: 408-924-2272
sjsu.edu/research

To: Dr. Masaaki Tsuruike
Department of Kinesiology
San Jose State University
One Washington Square
San Jose, CA 95192-0054

From: Pamela Stacks, Ph.D.
Associate Vice President
Office of Research

Date: July 27, 2017

The Human Subjects-Institutional Review Board has approved your request for an extension for the use of human subjects in the study entitled:

"Effect of Change of Knee Joint Angle on the EMG Activity of Hamstrings Muscles during OKC and CKC Exercise"

This approval is contingent upon the subjects participating in your research project being appropriately protected from risk. This includes the protection of the confidentiality of the subjects' identity when they participate in your research project, and with regard to all data that may be collected from the subjects. The approval includes continued monitoring of your research by the Board to assure that the subjects are being adequately and properly protected from such risks. If at any time a subject becomes injured or complains of injury, you must notify Dr. Pamela Stacks, Ph.D. immediately. Injury includes but is not limited to bodily harm, psychological trauma, and release of potentially damaging personal information. This approval for the human subject's portion of your project is in effect until the end of the year, and data collection beyond July 27, 2018 requires an extension request.

Please also be advised that all subjects need to be fully informed and aware that their participation in your research project is voluntary, and that he or she may withdraw from the project at any time. Further, a subject's participation, refusal to participate, or withdrawal will not affect any services that the subject is receiving or will receive at the institution in which the research is being conducted. If you have any questions, please contact me at (408) 924-2479.

Protocol # F16079
